# Long-term improvement by ozone treatment in chronic pain secondary to chemotherapy-induced peripheral neuropathy: A preliminary report

**DOI:** 10.3389/fphys.2022.935269

**Published:** 2022-08-30

**Authors:** Bernardino Clavo, Delvys Rodríguez-Abreu, Saray Galván, Mario Federico, Gregorio Martínez-Sánchez, Yolanda Ramallo-Fariña, Carla Antonelli, Gretel Benítez, Dolores Rey-Baltar, Ignacio J Jorge, Francisco Rodríguez-Esparragón, Pedro Serrano-Aguilar

**Affiliations:** ^1^ Research Unit, Hospital Universitario de Gran Canaria Dr. Negrín, Las Palmas de Gran Canaria, Spain; ^2^ Chronic Pain Unit, Dr. Negrín University Hospital, Las Palmas de Gran Canaria, Spain; ^3^ Radiation Oncology Department, Hospital Universitario Dr. Negrín, Las Palmas de Gran Canaria, Spain; ^4^ Fundación Canaria Instituto de Investigación Sanitaria de Canarias (FIISC), Las Palmas de Gran Canaria, Spain; ^5^ Molecular and Translational Pharmacology Group, Universitary Institute for Research in Biomedicine and Health (iUIBS), University of Las Palmas de Gran Canaria, Las Palmas de Gran Canaria, Spain; ^6^ Instituto Universitario de Enfermedades Tropicales y Salud Pública de Canarias de la Universidad de La Laguna, Tenerife, Spain; ^7^ Research Network on Health Services in Chronic Diseases (REDISSEC), Instituto de Salud Carlos III, Madrid, Spain; ^8^ CIBER de Enfermedades Infecciosas (CIBERINFEC), Instituto de Salud Carlos III, Madrid, Spain; ^9^ Spanish Group of Clinical Research in Radiation Oncology (GICOR), Madrid, Spain; ^10^ Medical Oncology Department, Complejo Hospitalario Universitario Insular Materno-Infantil de Gran Canaria, Las Palmas de Gran Canaria, Spain; ^11^ Medical Oncology Department, Hospital Universitario de Gran Canaria Dr. Negrín, Las Palmas de Gran Canaria, Spain; ^12^ Scientific Advisor, Freelance, Ancona, Italy; ^13^ Network for Research on Chronicity, Primary Care, and Health Promotion (RICAPPS), Tenerife, Spain; ^14^ Servicio de Evaluación y Planificación del Servicio Canario de Salud (SESCS), Santa Cruz de Tenerife, Spain; ^15^ Red de Agencias de Evaluación de Tecnologías Sanitarias y Prestaciones del Sistema Nacional de Salud (RedETS), Madrid, Spain

**Keywords:** antioxidants, cancer survivorship, cancer therapy-induced side effects, chemotherapy-induced peripheral neuropathy, neuropathic pain, oxidative stress, ozone therapy, pain

## Abstract

**Background:** Pain secondary to chemotherapy-induced peripheral neuropathy (CIPN) can limit the administration of chemotherapy, cancer-treatment outcomes, and the quality of life of patients. Oxidative stress and inflammation are some of the key mechanisms involved in CIPN. Successful treatments for CIPN are limited. This report shows our preliminary experience using ozone treatment as a modulator of oxidative stress in chronic pain secondary to CIPN.

**Methods:** Ozone treatment, by rectal insufflation, was administered in seven patients suffering from pain secondary to grade II or III CIPN. Pain was assessed by the visual analog scale (VAS).

**Results:** All patients, except one, showed clinically relevant pain improvement. Median pain score according to the VAS was 7 (range: 5–8) before ozone treatment, 4 (range: 2–6) at the end of ozone treatment (*p* = 0.004), 5.5 (range: 1.8–6.3) 3 months after the end of ozone treatment (*p* = 0.008), and 6 (range: 2.6–6.6) 6 months after the end of ozone treatment (*p* = 0.008). The toxicity grade, according to the Common Terminology Criteria for Adverse Events (CTCAE v.5.0), improved in half of the patients.

**Conclusion:** This report shows that most patients obtained clinically relevant and long-lasting improvement in chronic pain secondary to CIPN after treatment with ozone. These observed effects merit further research and support our ongoing randomized clinical trial (NCT04299893).

## Introduction

Chemotherapy-induced peripheral neuropathy (CIPN) is one of the most relevant side effects of chemotherapy. Its presentation before the end of chemotherapy (more frequent for oxaliplatin and paclitaxel) can lead to the delay, reduction, or even interruption of the initially planned scheme of chemotherapy, with potential decreases in tumor control. On the other hand, chronic CIPN weeks or months after the end of chemotherapy can lead to persistent impairments in quality of life. CIPN happens in 70–100% of cancer patients after treatment with platinum-based drugs and 19–85% of cancer patients after different neurotoxic chemotherapies (Zajaczkowska et al., 2019). A systematic review and meta-analysis showed a CIPN prevalence of around 2/3 within the first month of the end of chemotherapy with a progressive decrease until 1/3 at 6 months or later (Seretny et al., 2014). Unfortunately, there are no clinically relevant approaches for preventing CIPN and/or treating established CIPN, except for the limited effect described for duloxetine in the treatment of CIPN pain (Smith et al., 2013; Loprinzi et al., 2020; Bae et al., 2021; Zhang, 2021). The development and evaluation of novel strategies to mitigate and manage the chronic side effects of cancer therapy (CIPN included) have been established as urgent areas of research by the American Society of Clinical Oncology (ASCO) (Markham et al., 2020).

The pathomechanism by which chemotherapeutics damage nervous system structures and cause CIPN is multifactorial and involves chronic oxidative stress (OS) and neuroinflammation (Di Cesare Mannelli et al., 2012; Starobova and Vetter, 2017; Shim et al., 2019; Zajaczkowska et al., 2019; Salat, 2020; Bae et al., 2021). Based on those processes, we previously described how ozone can modulate OS and proinflammatory cytokine production as potential mechanisms of action in the prevention and improvement of different chemotherapy-induced toxicities (Clavo et al., 2019) and in CIPN (Clavo et al., 2021a).

The aim of this report was to show our preliminary experience using ozone treatment (O_3_T) as an adjuvant treatment in the palliative management of patients with chronic pain secondary to CIPN.

## Materials and methods

Between June 2019 and August 2021, 18 patients with different CIPN symptoms were submitted from Clinical Oncology and Radiation Oncology Departments to our multidisciplinary Chronic Pain Unit to evaluate complementary treatment with O_3_T. Some patients with mild symptoms were not treated with O3T, and some patients were treated with O3T because of numbness, tingling, or paresthesias without pain. Finally, seven patients were treated with O_3_T because of chronic and painful grade II or III CIPN. They were two males and five females, with a median age of 49 years old (between 36 and 73 years old). Compassionate ozone treatment in our hospital was assessed by the Health Care Ethics Committee, and this study was approved by the Provincial Research Ethics Committee of Las Palmas, Spain (Ref 2019-288-1). Informed written consent was obtained from all patients. See details in Table 1.

**TABLE 1:** Clinical characteristics and patient-reported changes in numbness, tingling, pain, and toxicity grade.

#	VAS pre/post O_3_	CTCAE * pre/post O_3_	Number of O_3_T sessions	Diagnosis, chemotherapy, and relevant clinical characteristics
1	Hands 8/6	3/2	50^a^	- Uterine adenosarcoma. TX: S + RT + CT (Ifosfamide + Doxorubicin)
	Feet 8/6			- Relevant clinical data: Tramadol intolerance. TX: AAINEs (Dexketoprofen, Paracetamol), Amitriptyline, Lormetazepam, and Capsaicin
2	Feet 5/3.5	3/2	40	- Endometrial serous-papillary carcinoma. TX: S + RT + ChT (Cisplatin + Paclitaxel)
3	Hands 7/7	2/2	40	- Ovarian serous carcinoma + endometrial carcinoma
	Feet 7/7			S + RT + CT: Carboplatin + Paclitaxel (50% reduction was required)
4	Hands 3/1	2/2	12^b^	- Colon adenocarcinoma. TX: S + CT: 1st line: Capecitabine + Oxaliplatin + Bevacizumab (Oxaliplatin withdrawal was required). O_3_T during the 2nd line CT: Folinic acid + 5-FU + Irinotecan + Aflibercept
	Feet 6/4			- Relevant clinical data: Ibuprofen allergic. TX: Gabapentin, Amitriptyline, and Tramadol
5	Feet 8.5/6	2/2	40	- Colon adenocarcinoma. S + ChT (Folinic acid + 5-FU + Oxaliplatin; 75% reduction was required)
6	Hands 8/2	2/1	40	- Non-Hodgkin’s lymphoma of parotid. TX: S + RT + CT (Cyclophosphamide, Doxorubicin, Vincristine, and Prednisone)
	Feet 6.5/3			- Relevant clinical data: Metamizol and Dexketoprofen allergic. TX: Tapentadol, Amitriptyline, Citalopram, and Pregabalin. After O_3_T: withdrawal of Amitriptyline and Citalopram and reduction in Tapentadol (50%)
7	Feet 4/1.5	2/1	40	- Colon adenocarcinoma Stage IV. -S + CT: 1st line: Folinic acid + 5-FU + Oxaliplatin. 2nd line: Folinic acid + 5-FU + Irinotecan (reductions were required because of hematologic toxicity and neuropathy). O_3_T during the 3rd line CT: Folinic acid + 5-FU + Oxaliplatin (60% reduction) + Cetuximab
				- Relevant clinical data: previous Rheumatoid arthritis
	7 (5–8)	2 (2–3)		Median (range from 25 to 75%) pre O_3_
	4 (2–6)	2 (1–2)		Median (range from 25 to 75%) pos O_3_
	*p* = 0.004 **	*p* = 0.125 **		

F: Female. M: Male. O3: ozone. S: Surgery. RT: Radiotherapy. CT: Chemotherapy. VAS: Visual Analogue Scale. CTCAE: Common Terminology Criteria for Adverse Events v.5.0 (toxicity-gradation system according to the US, National Cancer Institute). 5-FU: 5-Fluorouracil.

*Grades of toxicity according to the CTCAE: grade 1: mild symptoms; grade 2: moderate symptoms, limiting instrumental activities of daily live (ADL); grade indicated. 3: severe symptoms, limiting self-care in ADL; grade 4: life-threatening consequences; urgent intervention.

^**^Exact significance Wilcoxon Rank test.

a Patient #1, the number of sessions was extended until 50 at the request of the patient, after the previous improvement.

b Patient #4, O_3_T was administered during a poorly tolerated second line CT, and O_3_T was finished after 12 sessions in 4 months because of the many interruptions in O_3_T

Pain, according to the Visual Analog Scale (VAS) ranging from 0 (no pain) to 10 (the worst imaginable pain), was assessed before O_3_T, at the end of O_3_T, and at three and 6 months after the end of O_3_T.

O_3_T was administered by rectal insufflation on an outpatient basis. Ozone was obtained from clinical-grade oxygen using two medical ozone generators (Ozonosan Alpha-plus^®^; Dr. Hänsler GmbH, Iffezheim, Germany, and Ozonobaric P, Sedecal, Madrid, Spain). The O_3_/O_2_ gas mixture provided by the device was administered *via* a rectal cannula using standard 60 ml syringes. The initial O_3_/O_2_ concentration (in µg/mL: µg of O_3_ per mL of O_2_) was 10 μg/ml, and it was increased by 5 μg/ml every two sessions until a final concentration of 30 μg/ml was reached. Typically, for each patient, the gas volume for insufflation started at 180 ml/session and was slowly increased in successive sessions (depending on patient tolerance of bowel bloating) up to a maximum volume of 300 ml/session if tolerated. The initially planned treatment consisted of 40 sessions over 4 months, with three sessions per week during the first 2 months and two sessions per week later, although the final number of O_3_T sessions could vary according to the clinical evolution.

The SPSS software package (version 15 for Windows) was used for statistical analyses. All data were described as median and range from 25 to 75%. Paired comparisons for quantitative variables were conducted with the exact significance Wilcoxon Rank test. Though more conservative than asymptotic tests, exact tests were used due to the small sample size. *p*-values of <0.05 were considered statistically significant.

## Results

The median duration of O_3_T was 17 weeks (range from 17 to 17), and the median number of O_3_T sessions was 40 (range from 40 to 40). One patient commenced with CIPN symptoms 10 months after the end of chemotherapy, and all the other patients commenced with symptoms before the end of chemotherapy. The median time from the initiation of symptoms to the commencement of O_3_T was 12 months (range from 10 to 48). The median follow-up after the end of O_3_T was 8 months (range from 6 to 20).

**FIGURE 1:**
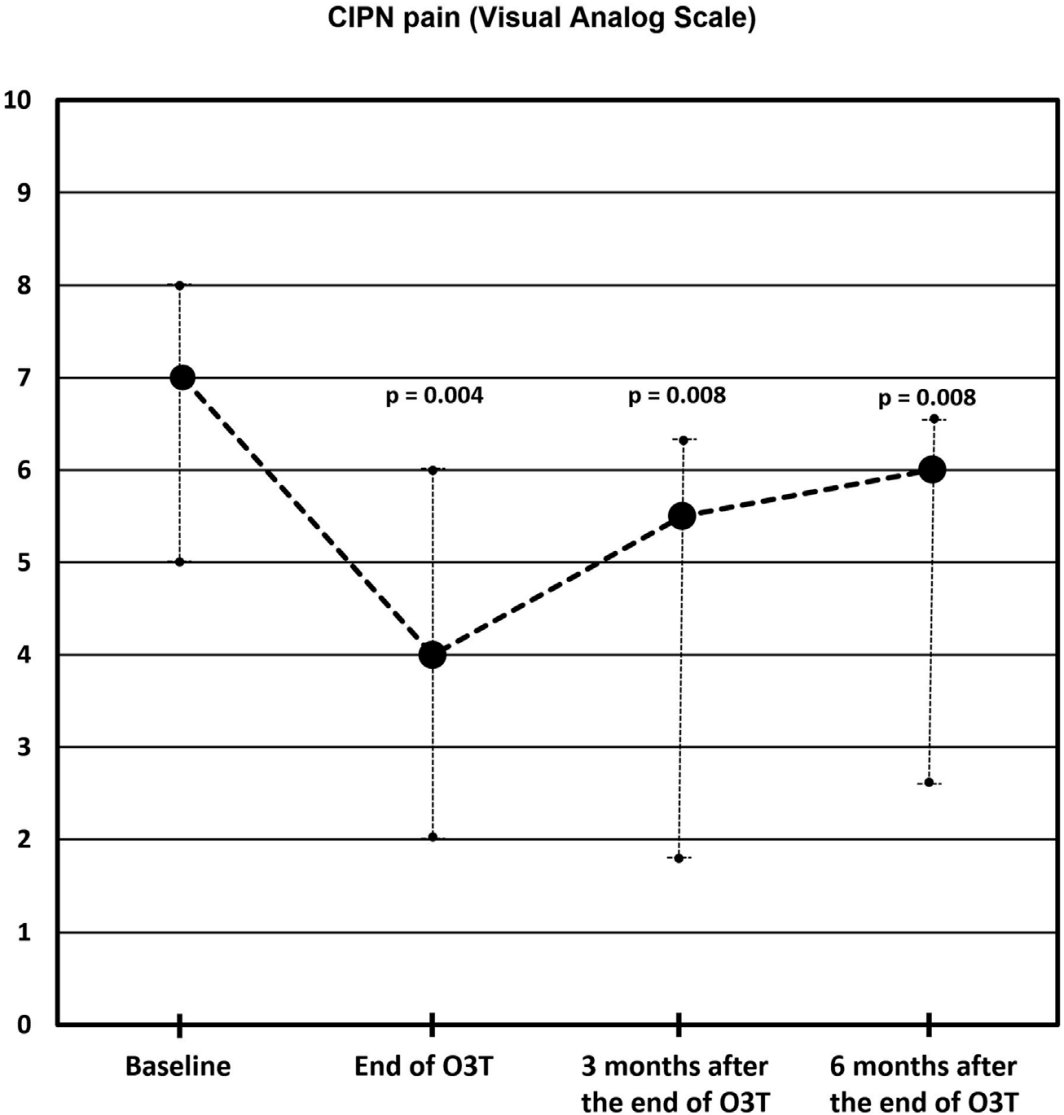
Chemotherapy-induced peripheral neuropathy (CIPN) pain according to the Visual Analog Scale (VAS). Compared to the baseline value, the median VAS pain score was significantly lower at the end of O_3_T (*p* = 0.004), 3 months (*p* = 0.008), and 6 months (*p* = 0.008) after the end of O_3_T (exact significance Wilcoxon Rank test).

Painful CIPN was independently assessed in 11 different locations, four hands and 7 feet (see Table 1). Six out of the seven patients (86%) reported pain decreases. Initial pain level and pain evolution over time were not necessarily equal in the different locations (hands and feet) for the same patient. In the 11 locations, the median VAS pain score was 7 (range from five to 8) before O_3_T, 4 (range from two to 6) at the end of O_3_T (*p* = 0.004), 5.5 (range from 1.8 to 6.3) 3 months after the end of O_3_T (*p* = 0.008), and 6 (range from 2.6 to 6.6) 6 months after the end of O_3_T (*p* = 0.008). See Figure 1.

The toxicity grade according to the CTCAE v.5.0 classification decreased in four out of the seven patients (no statically significant).

## Discussion

This preliminary report showed that adjuvant treatment with rectal O_3_T led to improvement in most patients with chronic pain secondary to CIPN, a frequent side effect of difficult management.

The pathogenesis and perpetuation of CIPN are multifactorial and may vary depending on the used drugs. Multiple involved mechanisms have been described as microtubule disruption, the alteration of calcium homeostasis and ion channel functions, DNA and/or mitochondrial damage, apoptosis, myelin sheath damage, and the induction of reactive oxygen species (ROS) by chemotherapy drugs with the further OS. At the same time, OS is linked to several of these factors, as well as to the induction of pro-inflammatory mediators that lead to immunological and inflammatory alterations (Di Cesare Mannelli et al., 2012; Starobova and Vetter, 2017; Shim et al., 2019; Zajaczkowska et al., 2019; Salat, 2020; Bae et al., 2021). ROS can also activate the apoptotic pathway in neuronal cells through mitochondrial pathway stimulation, including caspase activation (Zajaczkowska et al., 2019).

Recently, we described the potential role of O_3_T in the modulation of OS and inflammation in CIPN in detail (Clavo et al., 2019; Clavo et al., 2021a). Briefly, low/moderate ozone concentrations administered by rectal insufflation interact with unsaturated fatty acids from cell membranes in the intestinal mucosa. This produces a short and limited OS, with the generation of second messengers (e.g., aldehyde such as 4-hydroxynonenal (4-HNE) and H_2_O_2_), and the further and non-specific enhancement of adaptive antioxidant mechanisms, along with the transient upregulation of nuclear factor erythroid 2-related factor 2 (Nrf2). These effects are induced in a hormetic dose–response relationship. Therefore, the ozone effect is an “indirect effect” that does not follow a linear relationship. Significantly low ozone concentrations can have no effect, and significantly high concentrations can lead to undesired effects (Re et al., 2014; Galie et al., 2019; Tricarico and Travagli, 2021; Viebahn-Haensler and Leon Fernandez, 2021). In the same way, moderate physical exercise (not significantly low or high intensity) also induces temporal and soft increases in OS that lead to enhancements of antioxidant defense mechanisms and the regulation of OS (Kruk et al., 2019), with potential benefits for the prevention (Kleckner et al., 2018) and treatment (Kneis et al., 2019; Dhawan et al., 2020) of CIPN in randomized clinical trials. Both O_3_T and physical exercise share this hormetic dose-response based on the redox-activation of the nuclear factor erythroid 2-related factor 2 (Nrf2) (Re et al., 2014; Galie et al., 2019; Calabrese and Kozumbo, 2021; Viebahn-Haensler and Leon Fernandez, 2021). In addition, it has been demonstrated in animal models that O_3_T reduces the expressions of caspase-1-3-9 (Guclu et al., 2016) or normalizes mRNA caspase-1, caspase-12, and caspase-8 gene levels (Fuccio et al., 2009). Moreover, O_3_T inhibits autophagy of nerve root cells by decreasing cleaved caspase-3 expression, suppresses light chain 3B (LC3B) and Beclin one expression, decreases phosphodiesterase 2A and NF-kB p65 expression, and reduces nerve apoptosis by blocking the NF-kB signaling pathway (Wu et al., 2018).

Several other potential treatments based on the exogenous administration of antioxidants (e.g., acetylcysteine, amifostine, glutathione, vitamin E, and calmangafodipir) have been evaluated for the prevention or treatment of CIPN, although without conclusive results to date (Loprinzi et al., 2020). Overall, a potential explanation for the inconclusive results could be that those studies have been based on the increase of “only one” antioxidant or the use of a drug enhancing “only one step” in the overall and complex antioxidant defense mechanisms of the body. In contrast, as stated above, an appropriate ozone concentration can produce a limited (in magnitude and duration) and non-specific OS that is able to activate Nrf2 and induce an “overall” enhancement of the antioxidant defense mechanisms and the modulation of inflammatory cytokines all over the body. Once they are enhanced, some tissues could use them to palliate some pro-oxidative status (at the systemic or local level) as those associated with the side effects of radiotherapy (Clavo et al., 2005; Clavo et al., 2011; Clavo et al., 2013a), chemotherapy (Clavo et al., 2019), or CIPN. Additionally, we have described the beneficial effect of ozone treatment in different pain syndromes as refractory headaches (Clavo et al., 2013b), and refractory pelvic pain after cancer treatment (Clavo et al., 2021b), and a recent review has updated the evidence on ozone treatment in pain medicine, especially relevant in knee osteoarthritis and low-back pain (Hidalgo-Tallon et al., 2022).

There are no clinically relevant treatments for painful CIPN (Smith et al., 2013; Loprinzi et al., 2020; Bae et al., 2021; Zhang, 2021). The only agent that has appropriate evidence for the treatment of CIPN pain is duloxetine (60 mg once daily), based on a randomized clinical trial with 231 patients recruited by 10 centers (Smith et al., 2013; Loprinzi et al., 2020). The authors of this study reported a decreased pain of any amount of 59% in the duloxetine group and 38% in the placebo group. However, although the difference was statistically significant (*p* = 0.003), the magnitude of the improvement was modest, with a mean difference in average pain score of 0.73 (CI: 0.26, 1.20) between the duloxetine (1.06 score improvement) and placebo (0.34 score improvement) groups on a scale from 0 to 10 (Brief Pain Inventory-Short Form). These results show that further strategies for the management of chronic pain secondary to CIPN are required (Smith et al., 2013). This way, research on new approaches for the management of chronic side effects of cancer therapy (painful CIPN included) has been established as urgent area of research by the American Society of Clinical Oncology (Markham et al., 2020).

We highlight three aspects of the observed pain decreases in our study. First, most patients with painful CIPN reported long-term improvement after O_3_T (86%), even if two of them were treated with O_3_T during the second or the third line of chemotherapy (patients #4 and #7, respectively). This is more than twice the reported percentage (38%) of patients with improvement in the placebo groups of other studies on pain due to CIPN (Smith et al., 2013). Second, the magnitude of the observed effect (three points at the end of O_3_T) compared well with the effect described for duloxetine. Third, although the O_3_T effect decreased over time, it remained clinically relevant 6 months after the end of O_3_T, which was associated with decreased requirements for pain killer drugs and increased daily living activities. This protracted effect also compared well with the effect described for duloxetine study, which did not address long-term duloxetine treatment beyond 5 weeks, and half of its effect was lost 1 week after the treatment was finished. The longlasting effect of ozone in painful CIPN agrees with the effect of ozone described 9 months after the end of O_3_T in patients with chronic pelvic pain secondary to radiotherapy (Clavo et al., 2021b), and the effect described years after the end of O_3_T in patients with refractory hemorrhagic radiation-induced proctitis (Clavo et al., 2013a; Clavo et al., 2015) or refractory headache (Clavo et al., 2013b).

In our study, O_3_T by rectal insufflations was used instead of the systemic treatment through venous approach because the former has lower risks in cancer patients. Cancer patients with chronic and unresolved problems usually are frail patients. They already have the risk of several complications that could be erroneously attributed to O_3_T by a venous approach, but harder to be attributed to a rectal approach. On the other hand, after chemotherapy, the conventional venous access is difficult for most of these patients, and in that condition, it is not easy to think in a repetitive punction, several times per week, for several months, for blood extraction and reinfusion. Moreover, many cancer patients are using subcutaneous venous access, placed by the oncologist for chemotherapy administration, and these systems sometimes must be removed because of infection or thrombosis that we would not like to be attributed to ozone treatment.

The main side effect of O_3_T was found to be meteorism and bowel bloating secondary to gas insufflation, which usually disappeared after gas release by the patient and matches the results of previous reports (Clavo et al., 2013a; Viebahn-Hansler et al., 2016). The gas volume for rectal insufflation started at 180 ml/session and was slowly increased in successive sessions according to patient tolerance until 300 ml (if possible) or a well-tolerated volume. The planned maximum volume of 300 ml/session (total amount of 9,000 µg of O_3_ by session) was administered in all patients except three (patients #3, #4, and #6). Patient #3 was treated with 300 ml most days, although volume was between 240 and 300 ml during the full treatment depending on the feeling of abdominal distension. In patient #4, O_3_T was administered during the second-line chemotherapy, which produced diarrhea and chemotherapy interruptions, and only 120 ml of gas was insufflated in the ozone sessions. Patient #6 presented self-limited episodes of abdominal pain with/without diarrhea after two O_3_T sessions with a gas insufflation of 300 ml; then, O_3_T was continued with 240 ml of gas insufflation without further problems. No other side effects were associated with O_3_T in the patient study group.

This is a preliminary report with two relevant limitations. First, our study was a non-RCT with small sample size; however, they were patients refractory to conventional treatment after a median time of 12 months before the commencement of O_3_T. Second, the sample comprised patients with different tumor locations and different chemotherapy treatments, although most patients were treated because of colorectal cancer or gynecological tumors using platinum-based drugs.

Although this was a preliminary study, the magnitude and length of the observed effects and the limited therapeutical approaches for CIPN support further research with larger sample sizes, such as our ongoing randomized clinical trial (NCT04299893), to confirm our results.

## Conclusion

This preliminary report shows that most patients could obtain clinically relevant and long-term improvement in chronic pain secondary to CIPN after ozone treatment. The observed effects of ozone as an adjuvant treatment in the management of painful CIPN merit further research and support our ongoing randomized clinical trial.

## Data Availability

The raw data supporting the conclusions of this article will be made available by the authors, without undue reservation.
